# Supplementary material for: Development of a Nomogram to Estimate the 60-Day Probability of Death or Culling Due to Severe Clinical Mastitis in Dairy Cows at First Veterinary Clinical Evaluation
Source: Vet Sci. 2023 Apr 1;10(4):268. doi: 10.3390/vetsci10040268 (PMC10141895; doi:10.3390/vetsci10040268)
Supplement: Supplementary file 1 [file vetsci-10-00268-s001.zip › vetsci-2208522-supplementary.pdf]

veterinary sciences

MDPI

Supplementary material

Figure S1: Simple tutorial on nomogram usage

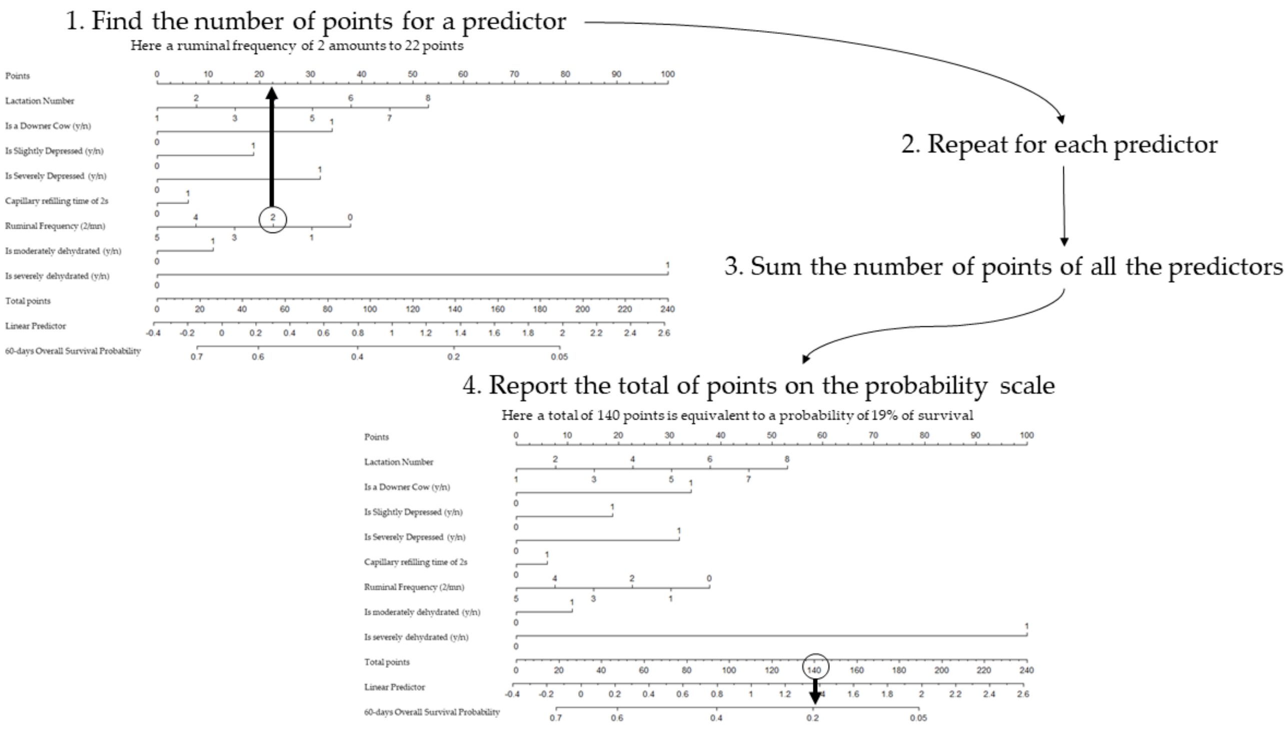

Figure S2: TRIPOD checklist

Section/Topic	Item		Checklist Item	Page
**Title and abstract**				
Title	1	D;V	Identify the study as developing and/or validating a multivariable prediction model, the target population, and the outcome to be predicted.	1
Abstract	2	D;V	Provide a summary of objectives, study design, setting, participants, sample size, predictors, outcome, statistical analysis, results, and conclusions.	1
**Introduction**				
Background and objectives	3a	D;V	Explain the medical context (including whether diagnostic or prognostic) and rationale for developing or validating the multivariable prediction model, including references to existing models.	2-3
	3b	D;V	Specify the objectives, including whether the study describes the development or validation of the model or both.	3
**Methods**				
Source of data	4a	D;V	Describe the study design or source of data (e.g., randomized trial, cohort, or registry data), separately for the development and validation data sets, if applicable.	3
	4b	D;V	Specify the key study dates, including start of accrual; end of accrual; and, if applicable, end of follow-up.	3
Participants	5a	D;V	Specify key elements of the study setting (e.g., primary care, secondary care, general population) including number and location of centres.	3
	5b	D;V	Describe eligibility criteria for participants.	3
	5c	D;V	Give details of treatments received, if relevant.	Figure S2
Outcome	6a	D;V	Clearly define the outcome that is predicted by the prediction model, including how and when assessed.	4
	6b	D;V	Report any actions to blind assessment of the outcome to be predicted.	4
Predictors	7a	D;V	Clearly define all predictors used in developing or validating the multivariable prediction model, including how and when they were measured.	4
	7b	D;V	Report any actions to blind assessment of predictors for the outcome and other predictors.	4
Sample size	8	D;V	Explain how the study size was arrived at.	3
Missing data	9	D;V	Describe how missing data were handled (e.g., complete-case analysis, single imputation, multiple imputation) with details of any imputation method.	5
Statistical analysis methods	10a	D	Describe how predictors were handled in the analyses.	5
	10b	D	Specify type of model, all model-building procedures (including any predictor selection), and method for internal validation.	5
	10c	V	For validation, describe how the predictions were calculated.	6
	10d	D;V	Specify all measures used to assess model performance and, if relevant, to compare multiple models.	6
	10e	V	Describe any model updating (e.g., recalibration) arising from the validation, if done.	
Risk groups	11	D;V	Provide details on how risk groups were created, if done.	
Development vs. validation	12	V	For validation, identify any differences from the development data in setting, eligibility criteria, outcome, and predictors.	
**Results**				
Participants	13a	D;V	Describe the flow of participants through the study, including the number of participants with and without the outcome and, if applicable, a summary of the follow-up time. A diagram may be helpful.	6-7
	13b	D;V	Describe the characteristics of the participants (basic demographics, clinical features, available predictors), including the number of participants with missing data for predictors and outcome.	7
	13c	V	For validation, show a comparison with the development data of the distribution of important variables (demographics, predictors and outcome).	
Model development	14a	D	Specify the number of participants and outcome events in each analysis.	7-9
	14b	D	If done, report the unadjusted association between each candidate predictor and outcome.	7-9
Model specification	15a	D	Present the full prediction model to allow predictions for individuals (i.e., all regression coefficients, and model intercept or baseline survival at a given time point).	9
	15b	D	Explain how to the use the prediction model.	9
Model performance	16	D;V	Report performance measures (with CIs) for the prediction model.	10-12
Model-updating	17	V	If done, report the results from any model updating (i.e., model specification, model performance).	
**Discussion**				
Limitations	18	D;V	Discuss any limitations of the study (such as nonrepresentative sample, few events per predictor, missing data).	13-14
Interpretation	19a	V	For validation, discuss the results with reference to performance in the development data, and any other validation data.	
	19b	D;V	Give an overall interpretation of the results, considering objectives, limitations, results from similar studies, and other relevant evidence.	13-14
Implications	20	D;V	Discuss the potential clinical use of the model and implications for future research.	13-14
**Other information**				
Supplementary information	21	D;V	Provide information about the availability of supplementary resources, such as study protocol, Web calculator, and data sets.	15
Funding	22	D;V	Give the source of funding and the role of the funders for the present study.	15

Table S1: Descriptive statistics by survival status, and univariable analysis of treatments of dairy cows coming from 124 herds in Québec, Canada with severe clinical mastitis in the 60-day period following a veterinary clinical evaluation. Statistical test was computed only between survival groups.

	N	Overall, N = 222[1]	Survival, N = 110[1]	Non-survival, N = 112[1]	p-value[2]
**Calcium**	215	114 (53%)	51 (49%)	63 (57%)	0.2
**Aminolean™**	215	2 (1%)	0 (0%)	2 (2%)	0.5
**Dextrose 50%**	215	10 (5%)	6 (6%)	4 (4%)	0.5
**Isotonic fluids**	215	20 (9%)	7 (7%)	13 (12%)	0.2
**Hypertonic fluids**	215	155 (72%)	80 (76%)	75 (68%)	0.2
**Oral fluids**	215	15 (7%)	8 (8%)	7 (6%)	0.7
**Anti-inflammatories**	218				0.6
No treatment		9 (4%)	3 (3%)	6 (5%)	
Aspirin		1 (1%)	1 (1%)	0 (0%)	
Dexamethasone		3 (2%)	1 (1%)	2 (2%)	
Flunixin		191 (88%)	97 (91%)	94 (85%)	
Ketoprofen		11 (5%)	5 (5%)	6 (5%)	
Meloxicam		2 (1%)	0 (0%)	2 (2%)	
Phenylbutazone		1 (1%)	0 (0%)	1 (1%)	
**Systemic Antimicrobial**	217				0.035
No treatment		5 (2%)	3 (3%)	2 (2%)	
Ampicillin		1 (1%)	0 (0%)	1 (1%)	
Oxytetracyclin		39 (18%)	20 (19%)	19 (17%)	
Penicillin		2 (1%)	0 (0%)	2 (2%)	
Penicillin & TMS[3]		7 (3%)	0 (0%)	7 (6%)	
Sulfadoxine Trimetoprim		163 (75%)	83 (78%)	80 (72%)	
**Intrammary Antimicrobial**	213				0.8
No treatment		27 (13%)	14 (13%)	13 (12%)	
Cefalak™		4 (2%)	1 (1%)	3 (3%)	
Erytho 36™		2 (1%)	2 (2%)	0 (0%)	
Homemade preparation		14 (7%)	7 (7%)	7 (7%)	
Pirsue™		4 (2%)	3 (3%)	1 (1%)	
SP 17900™		61 (29%)	31 (29%)	30 (28%)	
Spectramast LC™		101 (47%)	49 (46%)	52 (49%)	

[1]n (%),[2]Pearson's Chi-squared test; Fisher's exact test; [3] Sulfadoxine & Trimetoprim
